# Simulation and Experimental Study on Enhancing Dimensional Accuracy of Polycarbonate Light Guides

**DOI:** 10.3390/polym16223203

**Published:** 2024-11-19

**Authors:** Jiri Vanek, Martin Ovsik, Jan Hanzlik, Michal Stanek

**Affiliations:** Faculty of Technology, Tomas Bata University in Zlin, Vavreckova 5669, 760 01 Zlin, Czech Republic; vanek@utb.cz (J.V.); j_hanzlik@utb.cz (J.H.); stanek@utb.cz (M.S.)

**Keywords:** injection molding, polycarbonate, thick-walled parts, light guides, quality enhancement, process evaluation

## Abstract

This research investigates the adaptation of conventional injection-molding techniques for producing thick-walled polycarbonate optical components, specifically targeting their application in automotive light guides. With the automotive industry’s growing demand for reliable yet cost-efficient optical products, the study examines how traditional injection-molding processes can be refined to enhance dimensional accuracy and reduce defects. Simulations and experimental trials were conducted to evaluate the impact of critical process parameters, such as melt temperature, mold temperature, injection pressure, and gate design, on the overall quality of the final components. The results show that by carefully optimizing these parameters, it is possible to significantly reduce common defects like warpage, surface imperfections, and dimensional instability. This research highlights the potential of existing molding techniques to meet high industry standards while maintaining cost-effectiveness, offering valuable guidance for manufacturers aiming to produce high-quality optical components for demanding applications like automotive lighting.

## 1. Introduction

In today’s industrial landscape, injection-molded components play a crucial role, especially in the automotive industry, where design flexibility and high precision are paramount. While conventional injection molding typically favors uniform wall thickness for consistent flow and minimal defects, the rising demand for thick-walled optical parts like automotive light guides presents unique challenges. These parts must meet increasingly stringent specifications, especially regarding optical clarity and dimensional accuracy. This research focuses on the optimization of standard injection-molding techniques to achieve the best possible results in thick-walled applications, focusing on practical automotive light guide samples. Building on previous studies that primarily addressed baseline quality parameters and gating design for thick-walled polycarbonate parts, this work aims to extend the understanding by providing a systematic analysis of process parameters such as mold temperature, injection pressure, and melt temperature, examining their combined effects on minimizing deformations and sink marks. This study’s contributions lie in highlighting the feasibility of achieving high-quality optical performance in light guides using conventional methods, presenting findings that serve as practical guidelines for similar automotive applications.

In the evolving landscape of industrial manufacturing, injection molding plays a pivotal role in producing high-precision polymer components, particularly in sectors like the automotive and optical industries. Traditionally, injection molding has been optimized for thin-walled parts, where uniform wall thickness ensures optimal melt flow, consistent cooling, and reduced material shrinkage, thus minimizing defects [1,2]. However, the increasing demand for complex optical components, such as thick-walled lenses and light guides, has introduced significant challenges, including maintaining optical clarity, dimensional accuracy, and superior surface finish [3,4]. These challenges are particularly relevant in the automotive sector, where such components are vital for modern vehicle designs due to their functional properties, lightweight nature, and ability to meet advanced design requirements.

The production of thick-walled optical parts requires meticulous control over the injection-molding process, especially when addressing common issues like warpage, shrinkage, and internal stresses [5,6]. Although advanced techniques such as multilayer injection molding have been explored to tackle these issues [7], this study focuses on how conventional injection-molding methods can be optimized to achieve satisfactory performance for such demanding applications [8,9]. The research explores the influence of key parameters, including gate design, mold temperature, and pressure profiles, which are essential in improving the optical quality and dimensional stability of thick-walled components [10].

Gate design optimization has been identified as a crucial factor in ensuring uniform flow and minimizing defects such as sink marks, particularly in thick-walled lenses [11,12]. Techniques integrating multi-objective optimization strategies, such as Particle Swarm Optimization (PSO) combined with Back Propagation Neural Networks (BPNN), have shown promising results in enhancing the production of moderately thick optical lenses by balancing multiple conflicting objectives like warpage and surface defects [13,14]. Moreover, innovations in mold design, including the use of conformal cooling channels, advanced gating systems, and counter-pressure injection-molding techniques, have significantly improved product quality by controlling the cooling process and minimizing internal stresses [15,16].

Recent advancements in counter-pressure injection molding and precise mold temperature control have been effective in reducing birefringence, a common issue in thick-walled optical components, thereby enhancing the overall optical performance [17]. Additionally, gate design improvements have led to better flow behavior, which is critical in maintaining optical clarity and reducing surface imperfections [18,19]. This research seeks to bridge the gap between standard injection-molding practices and the high requirements for producing thick-walled optical components that meet both aesthetic and functional criteria, especially in automotive light guides. The findings extend beyond previous work by focusing specifically on the combined optimization of mold and melt temperatures with injection pressures in conventional settings to highlight feasible improvements for complex geometries.

Due to the growing complexity of modern optical designs and the stringent demands of automotive applications, understanding how standard injection-molding processes can be adapted to produce high-quality thick-walled parts is crucial [20]. The insights from this research will contribute significantly to the ongoing development of more efficient and precise manufacturing methods, offering a balanced approach between cost-effectiveness and quality for industries that rely heavily on optical components.

Standard injection-molding techniques, though traditionally optimized for thin-walled parts, can still achieve satisfactory results for producing thick-walled optical components, especially in applications where high geometrical and optical precision is not mandatory. Research has shown that by addressing common issues such as warpage and surface inconsistencies through innovations in gate design, mold temperature control, and multi-objective optimization, conventional methods can effectively meet the growing demand for high-quality automotive light guides and similar components. Although advanced techniques and specialized equipment may be necessary for more critical optical applications, this study underscores that optimized standard methods can still provide reliable solutions in high-demand industries, offering a practical approach to balancing cost-effectiveness with quality.

## 2. Materials and Methods

### 2.1. Overview of the Experiment

The experimental design and execution for this study were carefully tailored to meet the specific requirements for producing thick-walled light guides, which are crucial components in automotive lighting systems. The design of the test samples and injection molds was supported by simulation software, which was essential due to the challenging geometry of the light guide that opposed conventional design principles for plastic parts. Through these simulations, a detailed understanding of the molding process was achieved, allowing the determination of optimal boundary conditions for setting the technological parameters during injection molding.

To systematically evaluate the influence of various process conditions, including melt temperature, mold temperature, and pressure levels, the statistical method of Design of Experiment (DOE) was applied. This approach enabled an in-depth analysis of how different parameter combinations affect the dimensional stability and overall quality of the produced light guides. The results from these experiments were compared with the desired CAD model geometries using advanced 3D scanning techniques, allowing for precise measurement and statistical analysis of any deviations.

**Figure 1:**
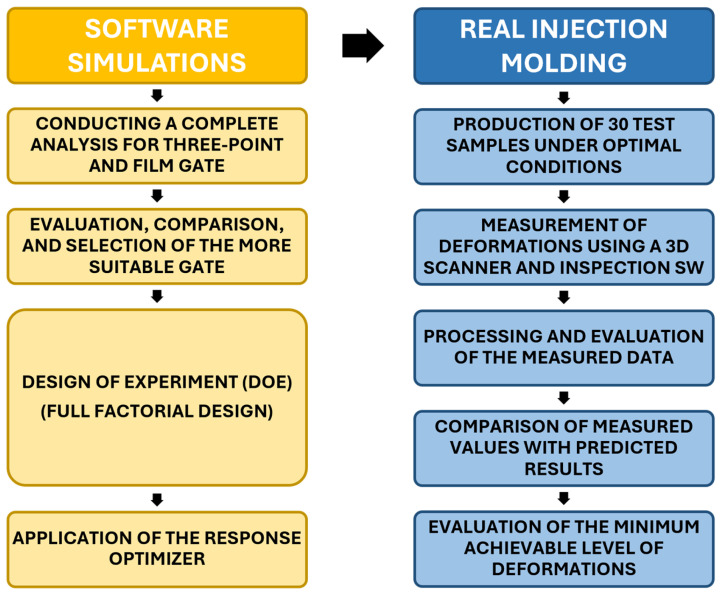
Schematic layout of the experiment.

The chosen methodology included three-dimensional modeling, injection-molding process simulations, the physical production of test samples, laser-based 3D surface scanning of the samples, and comprehensive data processing and evaluation. This approach not only enabled the identification and reduction of defects but also provided critical insights for optimizing conventional injection-molding techniques for complex thick-walled optical components. The experimental procedure is outlined schematically in Figure 1.

### 2.2. Materials

The injection-molded samples were produced using polycarbonate (PC) supplied by Covestro (Covestro AG, Leverkusen, Germany), marketed under the name Makrolon LED 2245. A natural transparent grade was selected, which can exhibit a faint yellowish hue when observed from the edge. This polymer is commonly used in optical and lighting applications due to its adaptability, high-light transmission, dimensional stability, and resistance to heat. The material also offers efficient light conductivity over a wide radiation spectrum, alongside maintaining stability in shape, temperature, and weather conditions, while also resisting yellowing. Prior to molding, the material was dried according to the manufacturer’s guidelines, which recommend a drying temperature of 120 °C for a duration of 3 h. The dried granulate was then pneumatically transferred to the injection-molding machine.

### 2.3. Software Tools and Technical Equipment

Various software tools and technical equipment were employed throughout this study. The designs for both test samples and injection molds were developed using CATIA V5 (Dassault Systèmes SE, Vélizy-Villacoublay, France), which facilitated the creation of all 3D models, assemblies, and manufacturing documentation. Two simulation software tools were utilized for analyzing the injection-molding process, chosen based on available university licenses. The combination of Moldflow 2024 (Autodesk, Inc., San Francisco, CA, USA) and Cadmould v17 (Simcon Kunststofftechnische Software GmbH, Würselen, Germany) allowed the study to leverage each software’s unique features and to compare the results of their simulations.

The test samples were produced using an Allrounder 470 E 1000-290 Golden Electric injection-molding machine (Arburg GmbH + Co KG, Loßburg, Germany), with parameters adjusted to suit the experimental requirements. The machine was supplemented by an Arburg Thermolift 100-2 dryer for pre-injection material drying and a Regloplas 150 Smart (Regloplas AG, St. Gallen, Switzerland) temperature-control unit, which ensured precise mold temperature management.

The surfaces of the manufactured samples were scanned using a Nikon MCAx30 laser 3D scanner (Nikon Corporation, Tokyo, Japan), with the collected data processed and evaluated using Polyworks 2023 (InnovMetric Software Inc., Quebec, QC, Canada). Lastly, statistical analysis was performed, and the results were interpreted using Minitab v22 (Minitab Inc., State College, PA, USA).

### 2.4. Design of Test Samples

The test sample used in this study was a functional thick-walled component designed as part of a rear automotive light assembly. The part’s dimensions were 80 × 50 × 75 mm, with the rib structure reaching a maximum thickness of 10 mm and the mounting base measuring 3 mm in thickness. The primary objective of this component was to ensure consistent light distribution, which was reflected in its design. The underside of the ribs was treated with an optical pattern specifically engineered to enhance light guidance and evenly spread the light across the desired areas.

### 2.5. Experimental Injection Mold

**Figure 2:**
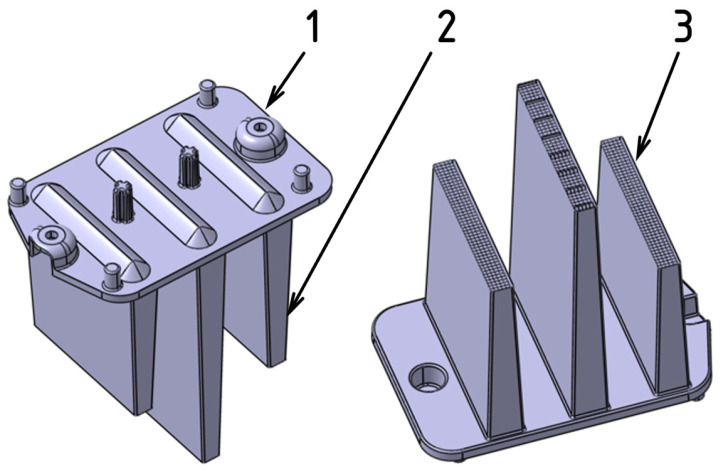
Three-dimensional model of the test sample: (1) mounting base; (2) optical rib; (3) optical pattern.

**Figure 3:**
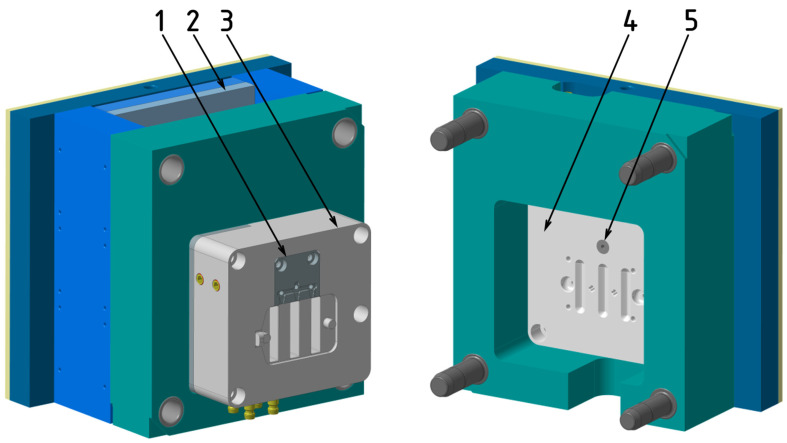
Injection mold for producing test samples: (1) interchangeable insert with gate; (2) ejector plates; (3) core insert; (4) cavity insert; (5) gate insert.

The production of all test samples was conducted using a single-cavity injection mold designed with interchangeable inserts that allowed for easy modification of the gate configuration (Figure 2). The mold’s gating system included a cold runner sprue and replaceable gate inserts. Temperature regulation was achieved using a system of drilled channels, with two channels located in the core and one in the cavity plate. The ejection of the molded part and runner was achieved through a mechanical system featuring both cylindrical and specially shaped ejectors, which engage the free ends of the ribs while simultaneously defining the optical pattern (Figure 3).

**Figure 4:**
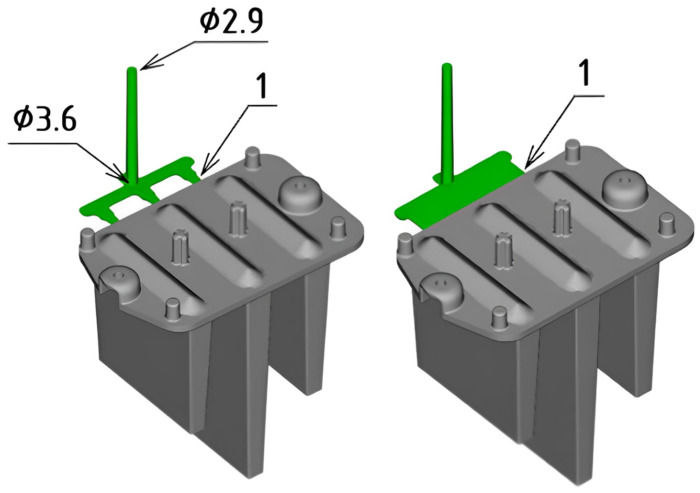
Variations of gate geometry (dimensions are in millimeters).

The injection molding of the functional light guides was conducted using two different gate geometries to evaluate which design provided the best cavity filling, aiming to minimize defects and reduce dimensional instability. Figure 4 illustrates the visual comparison of each gate system relative to the molded part. The first configuration featured a three-point gate with a thickness of 1 mm, while the second configuration used a film gate, also with a thickness of 1 mm.

### 2.6. Preparation of Injection-Molding Simulations

A key phase of the experiment involved evaluating the injection-molding process through simulations conducted using Autodesk Moldflow 2024 and Simcon Cadmould v17 software. The analysis focused on several critical aspects, including polymer melt flow, packing phase behavior, cooling efficiency, and deformation rates. This sequence allowed for comprehensive monitoring and assessment of each stage of the injection cycle, facilitating the identification and characterization of potential defects and undesirable phenomena, along with their root causes. The simulations were particularly useful in determining how gate system geometry, especially the gate location and design, impacts the filling process, leading to deformation or defects in the final molded parts. The insights gained from these evaluations helped identify the process parameters that significantly influenced the quality of the molded samples.

**Figure 5:**
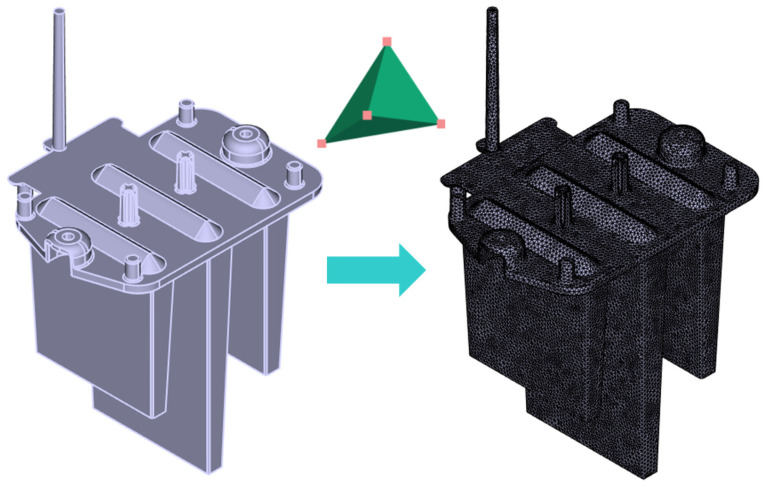
Imported 3D CAD model (**left**) and defined volumetric mesh (**right**).

The first step in preparing the simulations involved importing the 3D CAD model of the molded part, including the gate system, into Moldflow using the STEP format. Next, a 3D mesh was generated, dividing the surface and volume of the part into interconnected tetrahedral elements (Figure 5). These elements, composed of four surfaces, six edges, and four nodes, allowed for an accurate representation of thick-walled components and provided detailed results across the full thickness of the part. For consistency, the same mesh generated in Moldflow was also utilized in the Cadmould17 simulations, ensuring comparable results across both software platforms.

The mesh was initially created with a coarser element edge length, which was progressively refined through a mesh convergence analysis, leading to a final default edge length of 1 mm. This allowed for the identification of a converged deformation value without excessive computational cost. Larger edge lengths would not have adequately captured the detailed features of the part, potentially distorting the results, while further reducing the element size had minimal impact on accuracy but would have unnecessarily increased computational time and resource requirements. Since the Moldflow results were rounded to two decimal places, further precision in mesh refinement was deemed unnecessary. A manual mesh convergence study was conducted, as Moldflow lacks built-in tools for this analysis. Using element sizes smaller than 1 mm had minimal impact on the accuracy of the results, particularly in predicting deformations, but significantly increased simulation time. Therefore, a 1 mm mesh size was chosen as the best compromise between accuracy and computational efficiency.

The generated mesh was then evaluated against set criteria to ensure it met all simulation requirements (Table 1). The primary parameter examined was the aspect ratio between the longest and shortest edges of the tetrahedral elements, which should not exceed 30. The maximum recorded ratio was 17.48, with an average of 3.19. Additionally, the dihedral angle, which is the angle between the faces of the tetrahedra in the mesh, was maintained below 178 degrees, ensuring there were no excessively flat tetrahedra, which could reduce the accuracy in representing the 3D part. The mesh review also confirmed that there were no overlapping elements, disconnected nodes, or excessively high angles between surfaces, all of which could have compromised the accuracy or feasibility of the simulation.

**Figure 6:**
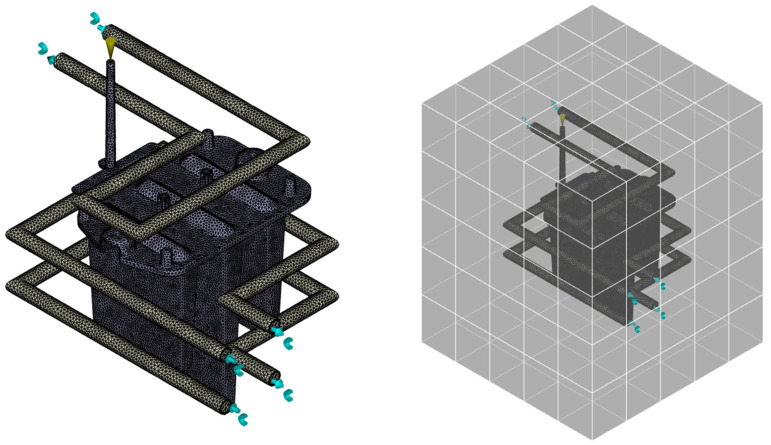
Volumetric mesh of cooling system and injection mold block.

The subsequent step involved modeling the cooling channels and mold block (Figure 6). The cooling system, including the positioning of inlets and outlets for the coolant, was based on the actual mold design. The core, cavity plates, and other mold elements were simplified into a solid block for simulation purposes. These geometries were also meshed, and their parameters were reviewed and adjusted accordingly. After this step, all remaining simulation conditions were set as summarized in Table 2.

Once the simulation was initiated, all necessary calculations were performed according to the predefined settings. The comprehensive Moldflow 2024 and Cadmould v17 analyses provided a range of critical outputs that visualized the processes occurring during each phase of the production cycle and enabled the evaluation of key quality indicators for the molded parts.

To determine the simulation parameters like filling time, packing pressure, and packing time in Table 2, we conducted a review of both manufacturer specifications for the material and initial test simulations to find suitable settings that reflect real-world injection molding conditions for thick-walled optical parts. These parameters were then adjusted to optimize flow and minimize defects, aligning with established guidelines and literature values for similar polycarbonate materials.

The injection pressure setting, marked as automatic, refers to the simulation software’s capability to dynamically adjust the pressure based on the selected flow rate and target fill time. This setting allows the software to maintain a consistent fill without manual intervention in pressure adjustments, providing controlled, real-time adaptation to maintain quality. This automated adjustment ensures an even material flow, reduces the likelihood of flow-related defects, and allows us to focus more closely on other parameters in the simulation setup.

The simulations specifically aimed to analyze filling time, the formation of weld lines, the solidified layer fraction for evaluating the effectiveness of the packing phase, shear rate, and shear stress at the mold wall. Additionally, temperature fields in the mold block and a thorough analysis of deformation and sink marks were included. The simulations were conducted for two types of gate configurations while maintaining consistent process parameters, allowing for a detailed examination of how gate geometry influences the injection process and the resulting part quality. The results from these analyses provide valuable information for stabilizing the injection-molding process and reducing defects.

### 2.7. Procedure of 3D Scanning and Dimensional Inspection

One method for comparing the deformation results obtained from simulations with those of the actual injection-molded part involves using 3D scanning technology. The surface of the produced sample was scanned using a laser 3D scanner, with the collected data then processed and compared to the reference CAD model’s geometry, which was designed to match the mold cavity dimensions. This technique proved to be highly effective in evaluating the dimensional accuracy and stability of the sample.

**Figure 7:**
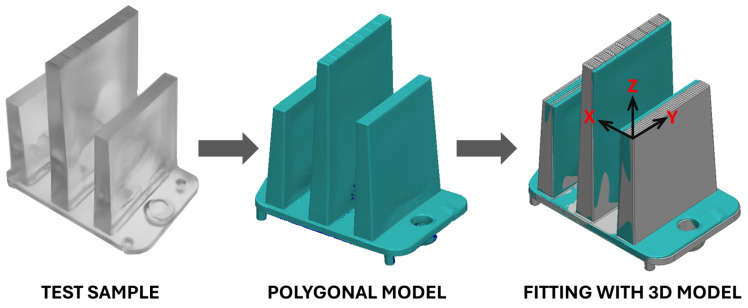
Procedure for comparing the geometry of the molded part with the 3D CAD model.

In a similar step, the Nikon MCAx30 PLUS laser 3D scanner was employed to scan the surface of the part, which was imported into the Polyworks 2023 software. The data were transformed into a meshed polygonal model, which was subsequently aligned with the reference CAD geometry using fitting algorithms. This evaluation process allowed for precise identification of geometric deviations between the scanned part and the CAD model. A color-coded map was used to visually represent these deviations, and from the point of view of the coordinate system, the maximum deviations were observed in the positive direction of the *x*-axis. Figure 7 illustrates the process used for this dimensional analysis.

**Figure 8:**
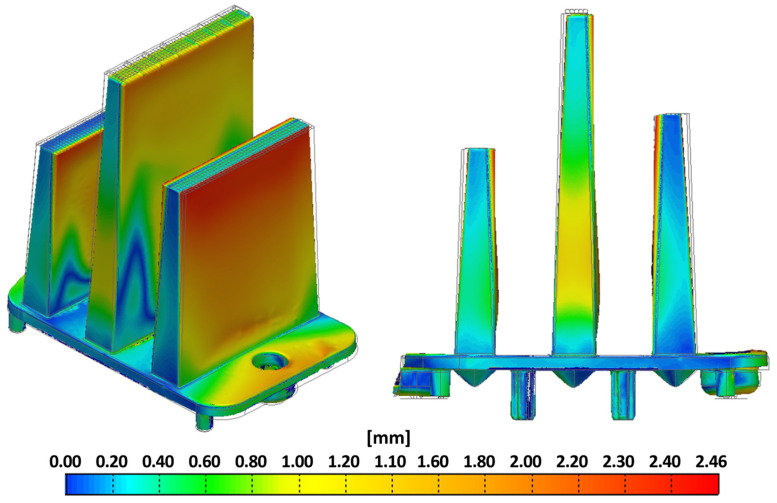
Color map representing the deviations of the product from the CAD model.

In the visualization (Figure 8), the blue areas represent the smallest deviations of the molded part from the ideal model, while the red areas highlight the largest deviations, with a maximum of 2.46 mm observed at the free ends of the optical ribs. These ribs deform similarly to what was predicted by the simulation results. In the Moldflow software, the maximum deformation was observed at the same location (free end of the right fib).

## 3. Results

### 3.1. Simulation Study

The simulation study aimed to evaluate how different gate designs influence key aspects of the injection-molding process, focusing on filling behavior, weld-line formation, frozen-layer distribution, shear stress, and temperature variations. Two gate configurations, film and three point, were compared under consistent process conditions to assess their impact on the shape and dimensional stability of the test sample. The analysis covered melt-front progression, weld lines, and frozen layers while examining the effects of shear stress and temperature inconsistencies on optical quality and residual stress. Sink marks were analyzed using Cadmould v17 to predict thickness variations in thick-walled parts more accurately. Results indicated that the film gate provided more uniform filling, fewer weld lines, and better packing efficiency, with potential for further improvement by increasing its flow cross-section.

#### 3.1.1. Fill Time

**Figure 9:**
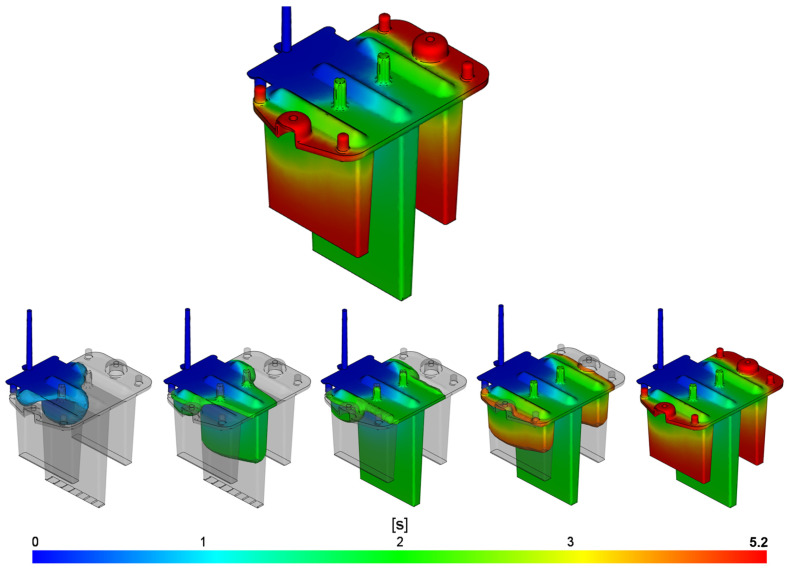
Fill-time result for the film gate.

The results of the filling-time analysis provided both graphical and numerical evaluations of the melt-front position within the mold cavity at regular intervals (Figure 9). This analysis allows for the determination of the time required to fully fill the cavity, including the gate system, and helps assess the uniformity of the filling process. Areas of the part that are filled at the same time are indicated by the same color. Dark blue represents the regions that are filled first, while red indicates the areas filled last. The color contours also illustrate the flow rate of the polymer; widely spaced contours suggest faster flow, while narrow contours indicate slower filling within the cavity.

The results indicate that the mold cavity was fully filled with melt in 5.2 s without any risk of short shots. The polymer flows through the sprue into the film gate, where it is distributed into the base of the part and then into the individual optical ribs. It is evident that a desirable fountain flow develops as the melt exits the gate. Monitoring the melt-front progression over time reveals an uneven filling pattern, with areas closer to the gate filling first because the melt does not need to travel as far as it does to reach areas further from the gate. The central rib of the part fills first at 2.8 s, while the side ribs and edges of the base are filled toward the end of the filling phase at 5.2 s. This uneven filling can contribute to the formation of unwanted weld lines.

**Figure 10:**
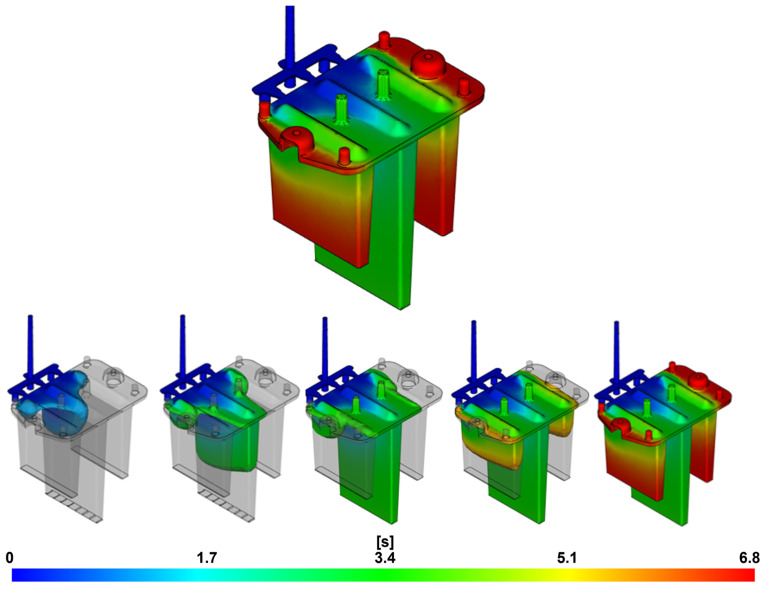
Fill-time result for the three-point gate.

A similar simulation was performed with the same process parameters but using an alternative three-point gate design (Figure 10). The visualization suggests even more uneven cavity filling compared to the previous film gate design. The melt primarily flows through the central gate, with a more significant filling of the side gates occurring around 3.4 s. By this time, the central rib is already filled, and the melt then flows into the side ribs and the remaining space in the base of the part. Complete filling is achieved at 6.8 s, as the three-point gate presents more flow resistance than the film gate design. These findings indicate a higher risk of dimensional instability and quality issues in the part produced with the three-point gate.

#### 3.1.2. Weld Lines

**Figure 11:**
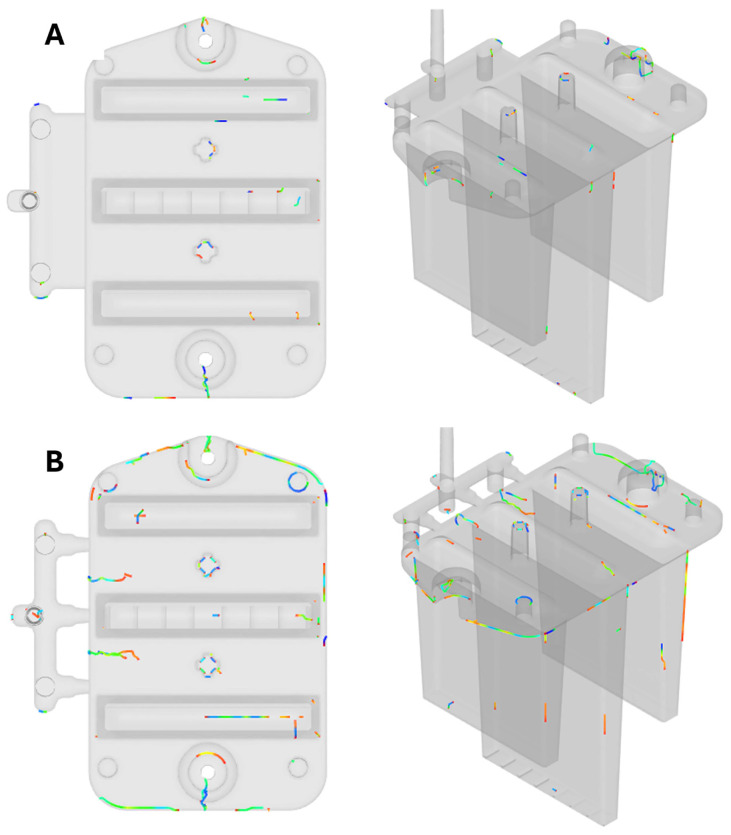
Result of weld-line occurrence: (**A**) film gate; (**B**) three-point gate.

The result shown in Figure 11 indicates the likely locations of weld lines in the molded part. This result is generated at the end of the cavity-filling phase. Weld lines occur when two or more melt fronts meet, leading to a local reduction in mechanical properties and creating surface defects. In optical parts, weld lines can significantly affect optical performance and cause visual defects, making the part less acceptable. The severity and quality of the weld line are influenced by factors such as the type of material being injected, the temperature of the converging melt fronts, the length of their common flow path, the gate system design, and the selected process parameters. For more complex geometries, it is practically impossible to completely avoid weld lines, but efforts should be made to minimize their impact.

The results indicate that a higher number of weld lines are present in the part filled using the three-point gate system, as the melt flow is divided into three streams during its passage. This leads to greater filling inconsistencies compared to the film gate, which shows fewer weld lines and appears more suitable for this part in terms of minimizing the formation of such defects.

#### 3.1.3. Frozen-Layer Fraction

**Figure 12:**
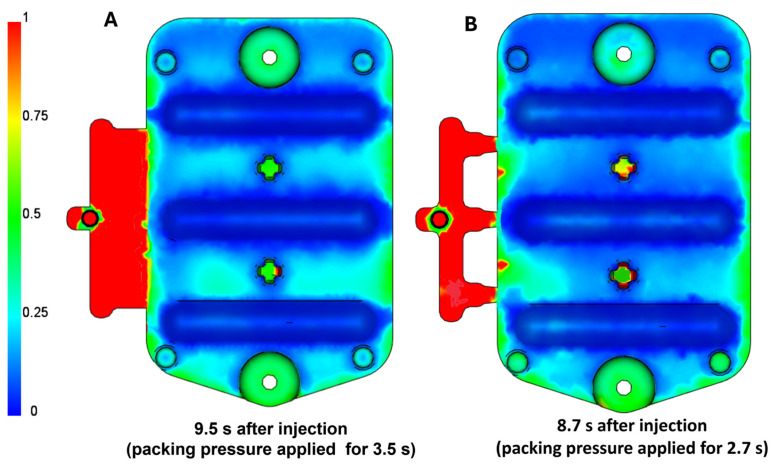
Visualization of the frozen-layer fraction result: (**A**) film gate; (**B**) three-point gate.

The result visualized in Figure 12 represents the thickness of the solidified layer of the injected material as a fraction of the total part thickness. These values range from zero to one, where higher values (shown in red) indicate a thicker frozen layer, greater resistance to flow, and a thinner or more restricted flow of molten polymer. The material is considered solidified when the temperature drops below the glass-transition temperature (145 °C). During the filling phase, the solidified layer should maintain a consistent thickness in areas with continuous flow, as heat losses to the mold walls are balanced by the incoming hot melt. When the flow stops, heat losses dominate, leading to a rapid increase in the thickness of the frozen layer, which significantly affects flow resistance. Viscosity rises exponentially as the temperature drops, and as the frozen layer thickens, the thickness of the flowing layer decreases.

The packing phase remains effective only until the gate or other thin-walled areas of the part completely solidify. The results show that, for the film gate, the packing phase is effective for approximately 3.5 s, whereas for the three-point gate, it is only 2.7 s. Due to the large volume and thick-walled nature of the part, there is a risk of significant deformation due to uneven shrinkage. Therefore, it is desirable to maximize compensation for volumetric shrinkage by continuing to feed additional melt into the mold cavity. The film gate allows for a longer effective packing time and more uniform cavity filling, resulting in reduced shrinkage and deformation compared to the three-point gate.

#### 3.1.4. Shear Rate

**Figure 13:**
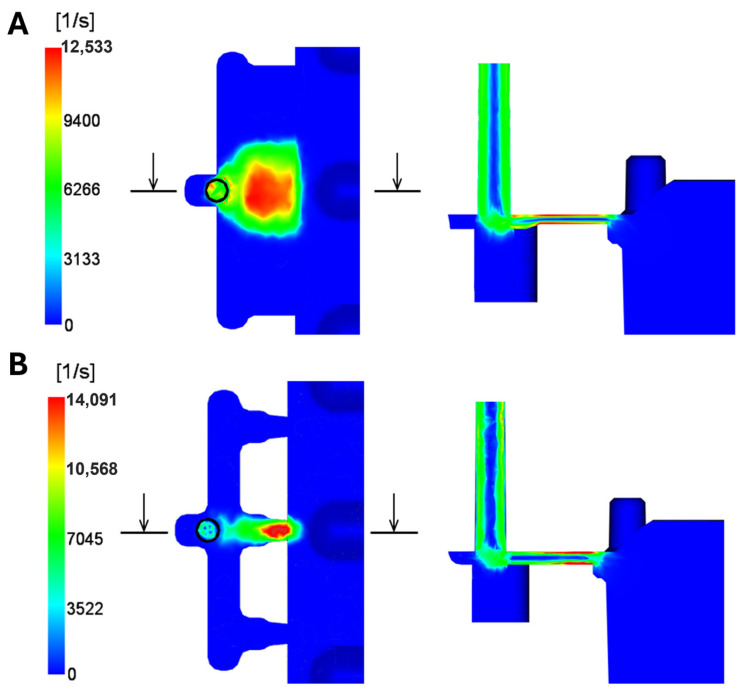
Representation of maximum shear rate: (**A**) film gate; (**B**) three-point gate.

The shear-rate results show the emerging values of the shear-deformation rate (velocity gradient across the section) within the mold cavity during injection and packing (Figure 13). The shear rate indicates how fast the layers of melt slide past each other. If this occurs too quickly, polymer chains can break, leading to material degradation, brittleness, and poor surface quality, which is unacceptable for optical parts.

During injection, the shear rate for the material in question should not exceed 40,000 s^−1^. The highest shear-deformation rates occur near the gate area in both gate system variants at the end of the filling phase when switching to the packing phase. The maximum value for the film gate reaches 12,533 s^−1^, while for the three-point gate, it reaches 14,091 s^−1^. These values are well within the allowable limit for the set process conditions.

#### 3.1.5. Shear Stress at Wall

**Figure 14:**
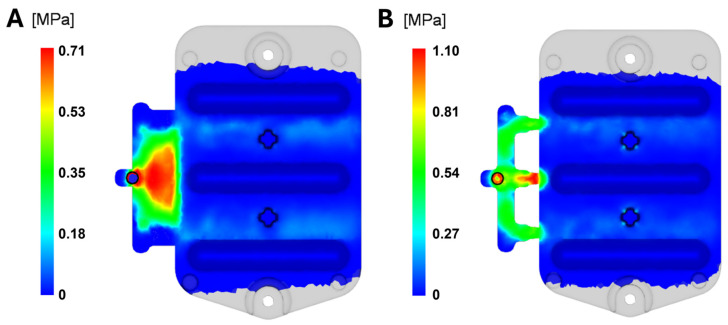
Shear stress at wall: (**A**) film gate; (**B**) three-point gate.

The results display the values of shear stress on the mold wall, occurring at the interface between the solidified layer of melt on the mold wall and the flowing melt (Figure 14). Although shear stress does not represent the actual residual stress in the part, it is closely related. It serves as an indicator of factors influencing the degree of melt orientation near the frozen layer. Materials with higher orientation tend to shrink more than non-oriented materials, so a high level of melt orientation near the mold wall compared to the center leads to increased residual stress. Elevated residual stress can result in part cracking during ejection or operation. For optical components, residual stress additionally causes inhomogeneous optical properties, which is why minimizing it is crucial.

The shear stress should remain below the maximum recommended value for the material, which is 0.5 MPa, as specified by the material manufacturer Covestro and available within the Moldflow database, which includes detailed processing conditions for this polymer material based on experimental data and standardized tests. For the film gate, this value peaks at 0.71 MPa at 4.2 s from the start of the cycle. For the three-point gate, the maximum reaches 1.10 MPa at 5.6 s. In both cases, the allowable limit specified in the material datasheet was exceeded. Although this effect is localized to the gate area, it can contribute to reduced quality of the final product since the melt may degrade as it passes through the gate system.

#### 3.1.6. Mold Temperature

**Figure 15:**
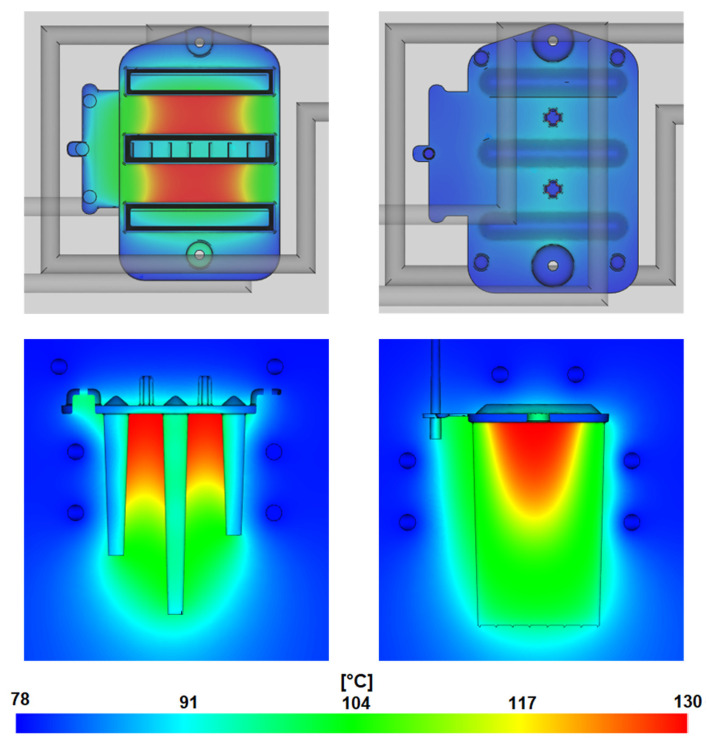
Mold temperature field.

The mold temperature results shown in Figure 15 represent the average mold temperature at the mold–cavity interface throughout the cycle, calculated using the Finite-Element Method (FEM). These results help identify temperature peaks and lows within the mold cavity and determine their impact on cooling time and resulting part deformations. For amorphous thermoplastics, the minimum and maximum cavity temperatures should ideally be within a 10 °C range of the target temperature. However, maintaining this criterion can be challenging in thick-walled parts. The smaller the temperature fluctuations on the mold cavity surface, the less likely they are to contribute to uneven cooling, subsequent part deformation, and prolonged cycle times. Temperature variations can be reduced by optimizing the placement of cooling channels, using conformal cooling, or employing high thermally conductive alloys in the mold’s construction.

In the simulation, the target mold temperature was set to the material manufacturer’s recommended value of 100 °C as specified in the Covestro material data provided by Moldflow. The results show that the maximum temperature between the thick ribs reaches approximately 130 °C, while local minima in the thin-walled outer edges of the part’s base show a temperature of only 80 °C. These temperature inconsistencies could be responsible for most of the deformations. Excess heat accumulates between the optical ribs because it is not effectively removed, as cooling channels are not present in this critical area.

#### 3.1.7. Deflection

In this analysis, the overall deformation magnitude in all directions was evaluated, considering differences in shrinkage rates and varying cooling intensities. The results are displayed using a color scale, where red indicates the most deformed areas of the part and blue represents the least deformed areas. The deformation size can also be compared against the outline of the undeformed reference CAD model of the part. The primary cause of the deformation is the unevenly distributed temperature field in the mold cavity and the varying shrinkage levels of the polymer due to the irregular wall thickness of the part.

**Figure 16:**
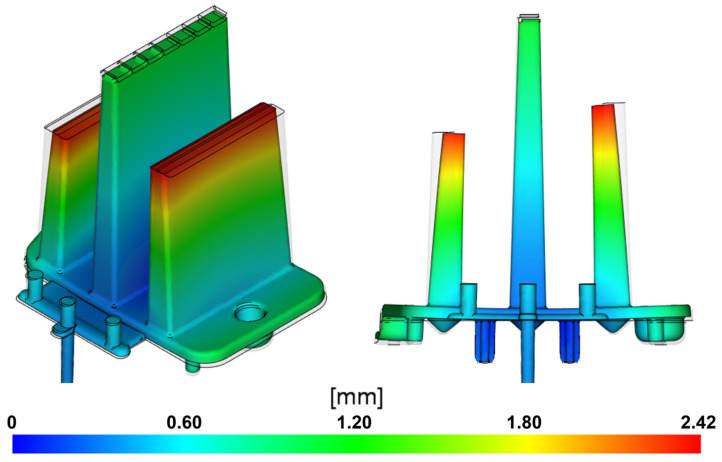
Visualization of deflection occurring when using a film gate (scale factor 2).

The visualization in Figure 16 shows that the deformations mainly occur as the side ribs bend toward the central rib. In the area of the optical pattern on the surface of the free ends of these ribs, the global deformation reaches a maximum of 2.42 mm. This deformation could negatively impact the intended optical properties of the part, affecting the uniformity of light distribution and causing distortion or refraction of the transmitted light. Additionally, the overall design of the product would be visibly altered, preventing it from fulfilling its intended aesthetic function. The mounting base of the part bends by 0.9 mm in the direction of the side rib deformation relative to the reference model, which could cause complications during assembly or even prevent this component from fitting correctly with other elements of the headlight assembly.

**Figure 17:**
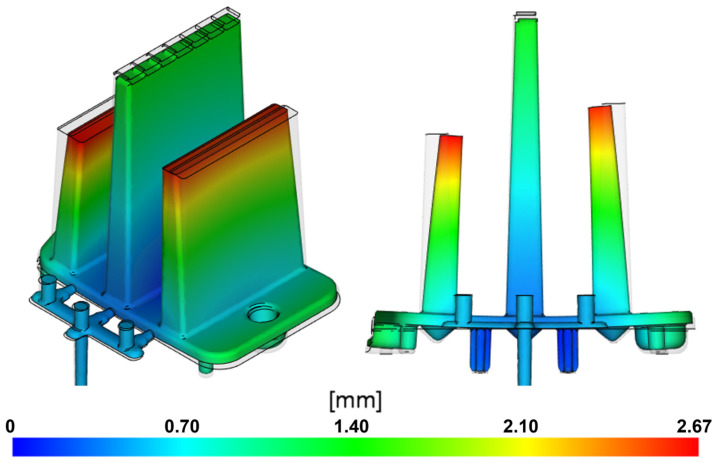
Visualization of deflection occurring when using a three-point gate (scale factor 2).

Similar results can be observed in the injection-molding simulation using a three-point gate (Figure 17). However, the overall deformations are greater, likely due to the limited effectiveness of the packing phase, as the point gate solidifies earlier than the film gate. This reduces the amount of melt delivered, along with the pressure compensating for volumetric changes. The increased deformation is also influenced by the less uniform cavity filling, which causes unstable flow and temperature conditions. The deformation peaks are again found at the ends of the optical ribs, with a value of 2.67 mm. At the mounting base, deviations from the reference model reach approximately 1.15 mm.

#### 3.1.8. Sink Marks

In this case, Cadmould v17 software was used specifically to analyze sink marks, as it is better suited for evaluating changes in the thickness of thick-walled parts. The standard software might provide misleading results when analyzing sink marks and thickness changes in such parts. These programs typically assess sink marks based on the distribution of volumetric shrinkage and part geometry, designed primarily to detect sink marks under ribs or in areas with localized increases in wall thickness. They are not optimized for evaluating sink marks across large volumes. For purely thick-walled components, the software may not accurately detect significant shrinkage due to its calculation method. On the other hand, Cadmould v17 is capable of predicting thickness changes in thick-walled parts, even in areas with substantial material volume.

**Figure 18:**
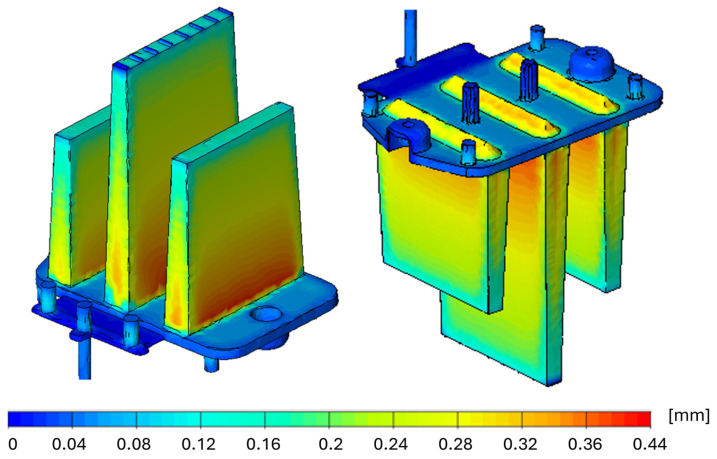
Visualization of sink marks occurring when using a film gate.

Therefore, an additional simulation was conducted to analyze the distribution and depth of sink marks (Figure 18), focusing on local thickness shrinkage in areas with high material accumulation, leading to differential volumetric shrinkage. Uneven cooling of the melt also contributes to this phenomenon. These inconsistencies manifest as shape and dimensional deviations, with the maximum sink mark depth in the thickest sections of the optical ribs reaching 0.44 mm when considering the film gate design.

**Figure 19:**
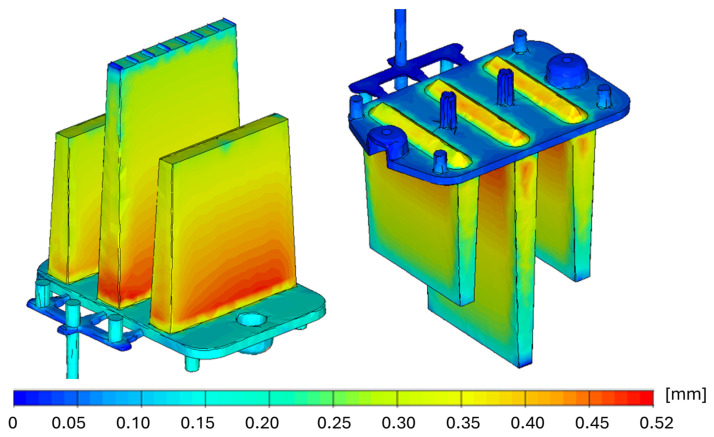
Visualization of sink marks occurring when using a three-point gate.

When the cavity is filled using the three-point gate, the sink-mark depth increases to 0.52 mm (Figure 19). It can be concluded that the deformations indicated in the previous results contribute significantly to the dimensional instability of the part, but the influence of sink marks should not be overlooked. Minimizing these defects is crucial for the proper functionality of the part. This can be achieved, for example, by ensuring more consistent heat removal from the mold cavity or optimizing the processing parameters.

#### 3.1.9. Summary of the Simulation Results

The injection-molding simulations for the thick-walled optical light guide were conducted for two gate designs under constant process conditions. These conditions were based on recommended values, both from material data and parameters suggested by the simulation software. The primary goal was to visualize and identify critical factors during the filling and cooling stages while evaluating the product’s shape and dimensional stability (Table 3). Deformations were simulated using a single software tool, focusing on how they aligned with the actual molded part dimensions. Additionally, sink marks were evaluated using Cadmould v17 software, which is better suited for predicting thickness variations in thick-walled parts, even in areas with substantial material concentrations.

Overall, the simulation results for the film gate configuration appeared more favorable regarding the analyzed aspects. The filling process was more consistent, the occurrence of cold weld lines was notably lower, the packing phase was more effective for an extended duration, and both shear stress and shear rate were reduced. The performance of the film gate could be further enhanced by increasing its flow cross-section, for example, by thickening it to 1.5 or 2 mm, which could potentially improve the results even further. In the case of the three-point gate, initial observations indicated that the central point gate predominantly filled the cavity due to uneven flow rates, leading to premature solidification at the side gates’ 1 mm-diameter orifices. The central orifice also experienced excessive shear due to high shear rates and stresses, which caused localized thermal degradation of the material.

To mitigate this issue in future designs, a balanced gate system is recommended. One approach could involve slightly enlarging the side gate orifices to help equalize flow rates across all gates, reducing the likelihood of premature solidification. Additionally, modifying the gate geometry to improve flow distribution and minimize shear stress in critical areas may further enhance performance. Due to these considerations, the next phase of experimentation will focus exclusively on optimizing a film gate, as it appears to be the most suitable option for achieving dimensional stability and minimizing defects in the tested samples. This will allow for a more effective approach to controlling the flow and reducing shear-related issues.

### 3.2. Reduction in Apparent Defects During Test Sample Preparation

After completing and evaluating the simulations, a test injection-molding process was conducted to observe the types of defects, flaws, and deformations that may actually occur. The objective was also to produce test samples without apparent defects, such as incomplete filling, cold weld lines, burn marks, yellowing, voids, or air bubbles, so that the surface of the samples could be scanned and the obtained data further processed.

The ranges for the process parameters in this study were determined based on material datasheet recommendations and initial experimental trials. The goal was to establish conditions where apparent defects like flow lines, incomplete filling, or discoloration would be avoided, enabling a focus on dimensional accuracy during test sample production.

#### 3.2.1. Specification of Tested Process Parameter Ranges

During initial molding trials, different levels of injection and packing pressures were tested to define an acceptable range that would accommodate variations in melt and mold temperatures while preventing surface defects. Packing pressure was set to 80% of injection pressure, which is slightly above recommended values, as a higher and extended packing phase was shown to reduce sink marks and ensure better dimensional stability.

Excessively high pressures led to visible defects like cracking, warping, and increased yellowing. Meanwhile, lower pressures caused voids, air traps, and poor surface finishes. Similarly, melt temperatures outside the 250–300 °C range and mold temperatures beyond 80–120 °C resulted in defects like premature solidification, poor flow, or surface degradation. After careful testing, the ranges of melt and mold temperatures were set to balance process stability and avoid major defects. Melt temperatures were kept between 250–300 °C, while mold temperatures were controlled within 80–120 °C. Injection pressure was held within 45–63 MPa, and packing pressure ranged between 36–50.4 MPa. This approach ensured that all test samples could be molded without visible surface defects like warping, voids, or discoloration. The chosen ranges shown in Table 4 enabled a focus on analyzing dimensional deviations while avoiding problems such as premature solidification or excessive material degradation. This setup allowed for a consistent analysis of the film gate’s performance while preparing the samples under optimized conditions.

#### 3.2.2. Identification and Reduction of Defects in the Injection-Molding Process

**Figure 20:**
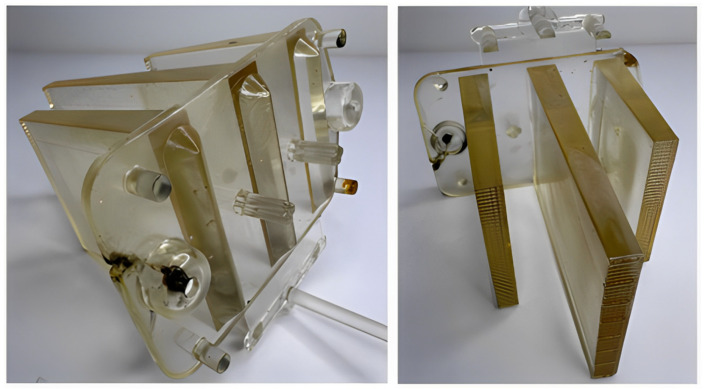
Yellowing of the material due to its thermal degradation.

The first major issue encountered during injection molding was the noticeable yellowing to browning of the material, which resulted from the thermal degradation of the polycarbonate caused by excessive melt temperature increases as the material passed through the gate (Figure 20). The level of shear stress likely exceeded acceptable limits due to high injection pressure and speed. This was also evident through a diesel effect, which led to a burnt area in the region of the cold weld that formed near the mounting hole at the base of the part.

**Figure 21:**
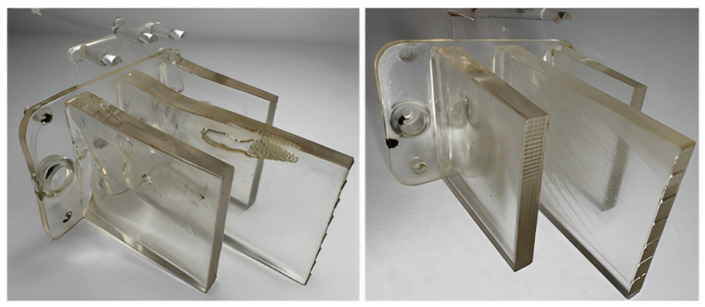
Warping of rib walls and air entrapment within the part’s volume.

Subsequently, both the injection pressure and speed were reduced, which resulted in a lower level of yellowing in the product. The cold weld line was less prominent compared to the previous case, and the diesel effect was less noticeable. However, this adjustment led to warping of the optical ribs and the mounting base, as well as shrinkage-induced voids and the formation of bubbles (Figure 21). Streaks appeared on the surface of the central rib in the flow direction, likely caused by the partially degraded material.

**Figure 22:**
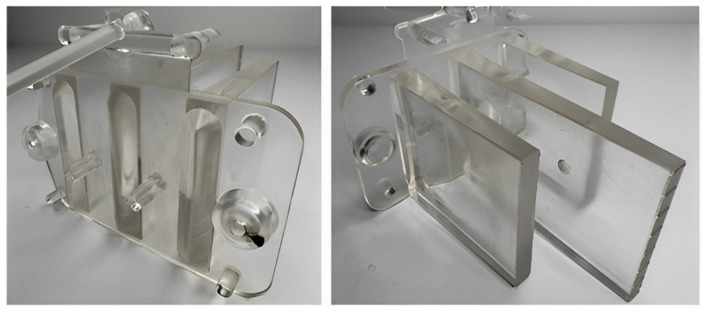
Test samples after partial defect reduction.

During the following trials, the melt temperature was further reduced, which, combined with lower pressure and injection speed, nearly eliminated yellowing and partially mitigated burn marks in the weld-line area. The size and frequency of entrapped air pockets were also limited, with only one noticeable bubble remaining in the central rib (Figure 22).

**Figure 23:**
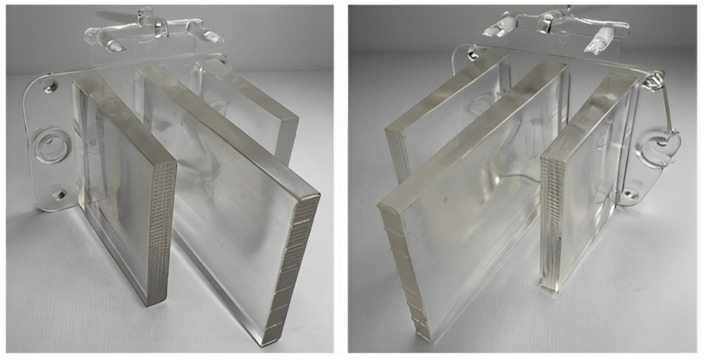
Test samples without apparent defects.

Another decrease in the previously mentioned parameters, combined with increasing the mold temperature, led to the complete elimination of the observed defects (Figure 23). The part produced under these optimized conditions showed no signs of yellowing, burn marks, bubbles, cold welds, or other visible surface defects. However, dimensional inconsistencies in terms of deformation and sink marks were still noticeable, allowing for the application of reverse engineering techniques. The surface of the part was scanned, and the obtained data were compared with the reference model.

### 3.3. Design of Experiment (DOE)

The experimental conditions were designed using Minitab 22 software, followed by data analysis that incorporated both simulation results and measurements obtained from laser scans of the injection-molded light guides. The goal of these experiments was to fine-tune the injection-molding parameters to achieve minimal deformation and sink-mark depth, particularly in the critical optical rib regions. The simulation findings helped identify significant process parameters and their interactions, leading to optimized settings that reduced deformation and sink marks. These conditions were subsequently validated by producing test samples and assessing actual geometric deviations. This approach allowed for the determination of optimal process conditions, minimizing defects and comparing the accuracy of simulation predictions with real molded parts.

The experimental conditions for the test sample molding were designed using a statistical Design of Experiment (DOE) approach to optimize the input parameters. A three-factor, two-level DOE setup was implemented, with one experimental repetition and a single block to ensure consistent results across simulations. The main factors examined included injection pressure, melt temperature, and mold temperature, with the primary output response being the maximal observed deformation of the prepared light guide.

The injection process was initially regulated by a flow rate of 14.7 cm^3^/s until the cavity reached 99% fullness. At that stage, control shifted to pressure control, applying injection pressures of either 45 MPa or 65 MPa. These pressures had a major influence during the packing phase, where the pressure was set to 80% of the maximum injection pressure, which equated to 36 MPa or 52 MPa. Each factor was tested at both minimum and maximum levels (Table 5), with the goal of systematically reducing deformations and sink marks, thereby ensuring the dimensional stability of the injection-molded test samples.

Table 6 outlines the input parameter design alongside the predicted deformations. A total of eight simulations were performed, with process parameters adjusted according to the specified ranges. The results obtained from these simulations were then utilized to assess the size of deformations, facilitating further analysis of the individual factors and their combined effects. This approach provided valuable insights into the influence of each parameter on the final outcome.

#### 3.3.1. Analyzing the Significance of Specific Tested Factors

**Figure 24:**
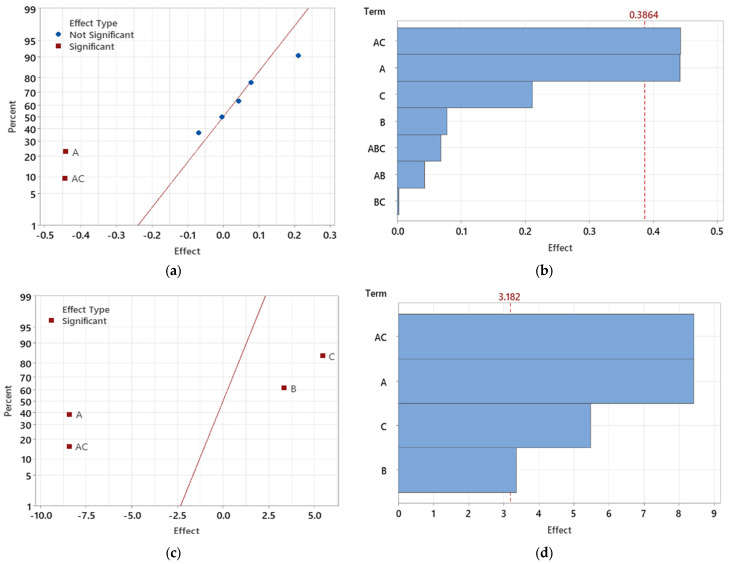
Effects of individual factors and their interactions on maximum deformation in light-guide samples: (**a**) Normal plot of effects for deformation analysis; (**b**) Pareto chart showing the magnitude of factor effects; (**c**) Normal plot of effects after reducing non-significant factors; (**d**) Pareto chart of standardized effects after factor reduction.

The normal plot of effects (Figure 24a) demonstrates the significance of individual parameters and their interactions with regard to the observed maximal deformations in the test samples. Significant factors, shown in red, deviate substantially from the zero axis, while the blue symbols represent insignificant factors closer to the axis. The interaction between injection pressure (A) and melt temperature (C) (AC) is identified as a significant contributor to deformation. Understanding these combined effects is critical to evaluating the overall outcome.

To further clarify the impact and significance of each factor, Pareto charts were generated for the light guide test specimens (Figure 24b). These charts help visualize and rank the effects, making it easier to identify the most influential factors. The red reference line represents the threshold for statistical significance, with factors above this line being significant. In this analysis, melt temperature (C) emerges as the most significant factor, followed by injection pressure (A), with their interaction (AC) also showing a substantial impact.

In this case, melt temperature (C) consistently proves to be a dominant factor in minimizing deformations, especially in thicker sections of the part. The interaction between injection pressure and melt temperature (AC) underscores the importance of adjusting both parameters to optimize dimensional stability. While mold temperature (B) is less statistically relevant, its interaction with injection pressure (AB) achieves statistical significance, demonstrating the need to consider combined effects in the optimization process.

The next step in the analysis involved the reduction of statistically insignificant factors. After the initial evaluation, non-significant effects were progressively eliminated, and the analysis was repeated until only factors that were significant at the α = 0.05 level remained. Following this reduction, the interaction between injection pressure and melt temperature (AC) became statistically significant (Figure 24c).

The Pareto chart of standardized effects (Figure 24d) provides a clearer understanding of the significance of these remaining factors. Injection pressure (A) and its interaction with melt temperature (AC) emerged as the most critical influences on maximum deformation in the light guide test sample. Additionally, while melt temperature (C) and mold temperature (B) remained statistically significant, they exhibited a comparatively lesser impact on the final deformation outcomes after the reduction process.

This refined analysis underscores the importance of controlling injection pressure and its interaction with mold temperature, as these factors dominate the influence on minimizing deformation. Though melt temperature and mold temperature are still statistically relevant, their role becomes less prominent, allowing for a more focused optimization strategy centered around the key influential parameters.

**Figure 25:**
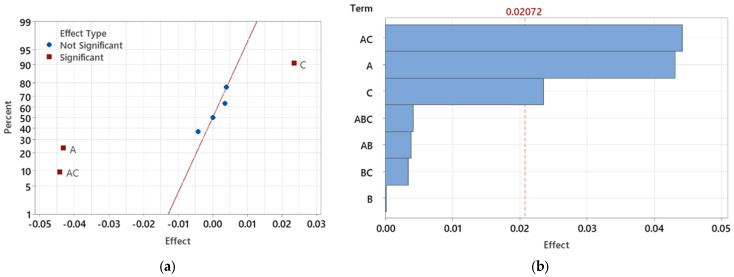

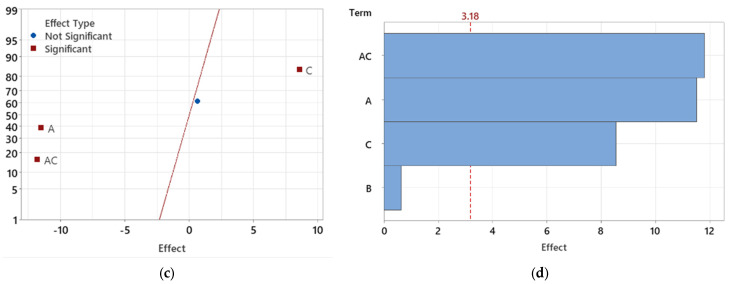
Effects of individual factors and their interactions on sink-mark depth in light guide samples: (**a**) Normal plot of effects for sink mark depth analysis; (**b**) Pareto chart showing the magnitude of factor effects; (**c**) Normal plot of effects after reducing non-significant factors; (**d**) Pareto chart of standardized effects after factor reduction.

For the analysis of maximum sink-mark depth, a similar Design of Experiments (DOE) methodology was applied, as previously done in the deformation study. In the initial evaluation, before reducing non-significant factors, it was found that melt temperature (C), injection pressure (A), and their interaction (AC) significantly affected sink-mark depth. This is illustrated in the Normal Plot of Effects (Figure 25a), where these parameters are furthest from the center, indicating their statistical significance. These findings are consistent with those from the deformation analysis, where melt temperature and injection pressure also played crucial roles.

The Pareto Chart (Figure 25b) further confirms these results by showing that the interaction between injection pressure and melt temperature (AC) had the greatest impact on sink-mark depth, followed by injection pressure (A) and melt temperature (C). While the same key factors influence both deformation and sink-mark depth, the magnitude of their effects varies. Notably, the interaction between injection pressure and melt temperature (AC) holds a more significant role in sink-mark depth than it did for deformation.

After reducing statistically insignificant factors, the updated Normal Plot of Standardized Effects (Figure 25c) and Pareto Chart (Figure 25d) indicate that injection pressure, melt temperature, and their interaction (AC) remain statistically significant. However, the results show that sink-mark depth is more strongly affected by interaction effects compared to deformation, suggesting a higher sensitivity to the combined effects of melt temperature and injection pressure.

In comparison to the deformation study, it is evident that both deformation and sink-mark depth are influenced by the same critical parameters. However, the sink-mark depth demonstrates a heightened sensitivity to the interaction between melt temperature and injection pressure. This emphasizes the importance of carefully optimizing these two parameters together to minimize sink marks in thick-walled parts like the light guide.

#### 3.3.2. Optimization

**Figure 26:**
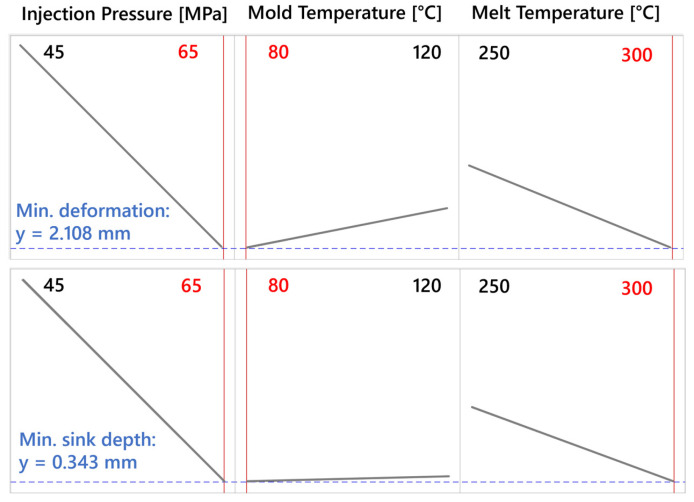
Optimized process conditions for minimizing deformation and sink-mark depth.

The optimization results presented in Figure 26 display two sets of optimized conditions: one aimed at minimizing deformation and the other focused on reducing sink-mark depth. Both optimizations were performed on the same light guide samples, emphasizing different quality responses based on the input factors.

For the deformation minimization, the optimization results show that the most significant factors influencing the reduction of deformation are injection pressure, mold temperature, and melt temperature. The results indicate that using the higher value of 65 MPa for injection pressure, a lower mold temperature of 80 °C, and the upper limit of melt temperature at 300 °C achieved the best result. The predicted minimal deformation under these conditions is 2.108 mm, which likely stems from maintaining a high-melt temperature that promotes better material flow and reduced internal stresses, balanced by a lower mold temperature to enhance dimensional stability during cooling.

When optimizing for sink-mark depth, the best result is achieved under similar conditions, with 65 MPa injection pressure, 80 °C mold temperature, and 300 °C melt temperature. The predicted minimal sink-mark depth under these conditions is 0.343 mm. The high-melt temperature likely improves flow and reduces premature solidification, which aids in minimizing sink marks in thicker sections of the part. At the same time, the higher injection pressure ensures that the material reaches all areas of the mold cavity, effectively reducing sink marks.

Both optimizations demonstrate the importance of maintaining a high injection pressure and melt temperature while keeping the mold temperature at a lower level. However, the specific results suggest that optimizing deformation and sink-mark depth requires careful balancing of these factors. The sink-mark depth optimization shows a slightly higher benefit from the interaction between injection pressure and melt temperature, while the deformation minimization shows a more pronounced dependency on the higher injection pressure to maintain dimensional stability across the light-guide test specimen.

#### 3.3.3. Overview of DOE Results

In this study, experiments were designed based on the Design of Experiment (DOE) methodology to analyze the influence of key process parameters on both deformation and sink-mark depth in the light-guide test specimens. The primary objective was to determine the optimal process conditions for minimizing these defects, focusing on injection pressure, melt temperature, and mold temperature as the main input factors.

For deformation, the DOE analysis identified melt temperature, injection pressure, and their interaction as the most significant factors impacting deformation. After reducing statistically insignificant factors, it became clear that injection pressure, along with its interaction with mold temperature, had the greatest influence on minimizing deformation. Although melt temperature and mold temperature remained statistically relevant, their effects were comparatively smaller in this context, as seen in Table 7.

Similarly, for sink-mark depth, the DOE analysis highlighted melt temperature, injection pressure, and their interaction as key influences. The interaction between melt temperature and injection pressure played an even more critical role in sink-mark depth compared to deformation, indicating a heightened sensitivity to these combined factors when addressing sink marks. After eliminating non-significant effects, injection pressure and melt temperature, as well as their interaction, remained the dominant factors affecting sink mark depth.

The results from the optimizer tool further support these findings. Table 8 provides a summary of the optimization results, showing that higher injection pressure and melt temperature, combined with a lower mold temperature, are the most effective in reducing both deformation and sink mark depth.

Overall, both deformation and sink-mark depth are strongly influenced by injection pressure, melt temperature, and their interaction. However, the relative importance of these factors differs depending on whether the deformation or sink-mark depth is being optimized. Injection pressure tends to have a slightly more pronounced effect on deformation, while sink-mark depth is more sensitive to the combined effects of injection pressure and melt temperature.

### 3.4. Statistical Evaluation of Deformations

At this stage, the research narrows its focus to studying deformations in the injection-molded light guides, as sink marks proved challenging to measure consistently. Specifically, identifying the precise locations of the maximum sink marks was problematic due to the complex geometry of the light guide and the relatively small contribution of sink marks to the overall dimensional stability of the part. In contrast, deformations exhibited a more pronounced effect on the functionality of the light guide, making them a more critical focus of this evaluation.

Furthermore, the DOE optimization settings, which effectively reduced deformations, also likely contributed to a reduction in sink-mark depth, as the process parameters applied were the same for both responses. Due to the overlap in process parameters, it can be reasonably assumed that improvements in deformation for this specific part will likely correspond with a reduction in sink-mark depth. However, this assumption is primarily relevant to the geometry and material characteristics of this particular light guide and should be considered cautiously for other applications.

**Figure 27:**
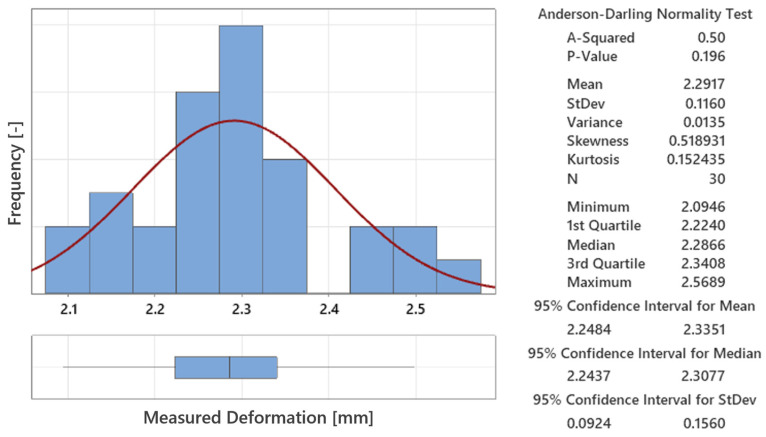
Distribution of measured deformations with a normality test confirming the data follows a normal distribution across all 30 test samples.

The following analysis presents a comparison between measured and simulated deformation data for the light-guide test specimens, based on the results from 30 samples. The histogram in Figure 27 shows the Anderson–Darling normality test for the measured deformations, with a *p*-value of 0.196, indicating that the data follow a normal distribution. The mean deformation of 2.2917 mm, with a standard deviation of 0.116 mm, suggests that the data are consistent, with deformations centered around this mean value. The box plot further confirms that there are no significant outliers, highlighting the stability of the injection-molding process across all 30 samples.

**Figure 28:**
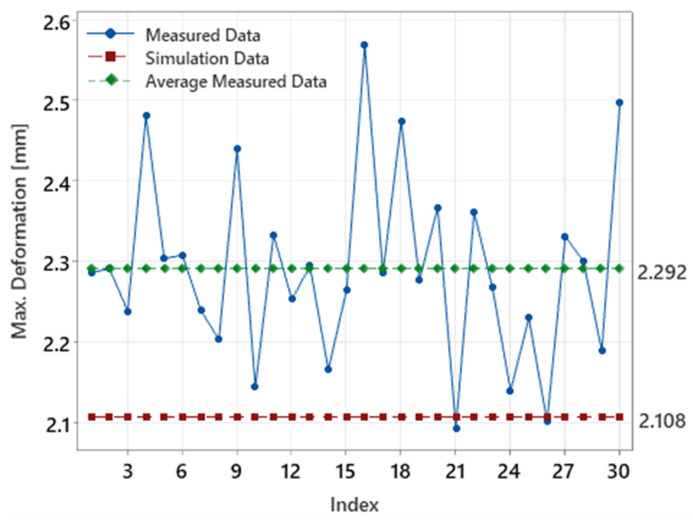
Comparison of measured deformations, simulation results, and average measured deformation across 30 test samples.

In Figure 28, the measured deformations (blue points) are compared to the predicted simulation results (red dashed line at 2.108 mm), with the average measured value marked by the green line at 2.292 mm. Although the simulation slightly underestimates the actual deformations by around 0.184 mm, this difference is not substantial. Due to the boundary conditions—such as process parameters, mold configuration, machine aspects, and material properties—this represents the lowest deformation achieved within the tested conditions. While the actual deformations are somewhat higher, the simulation still provides a strong approximation, showing that the predicted deformations align closely with the measured results.

**Figure 29:**
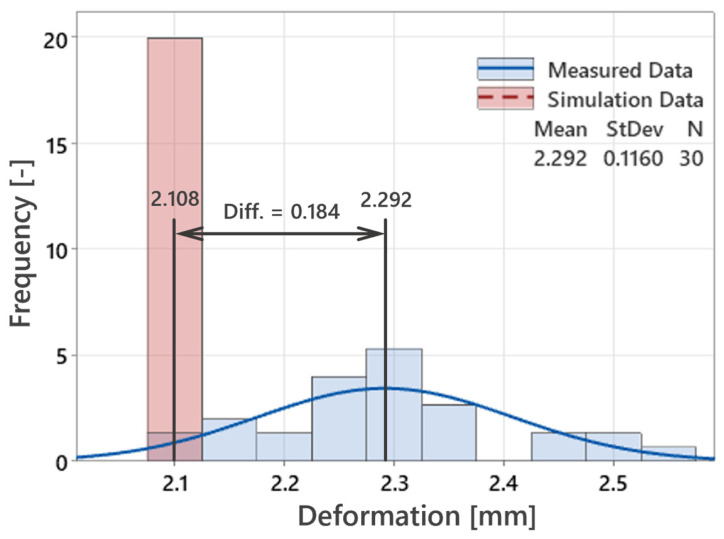
Comparison of the distribution of measured and simulated deformation values, showing the difference between the mean values.

Figure 29 illustrates the distribution of both measured and simulated deformation values. Most measured deformations fall within a broader range compared to the more concentrated simulation values. While the difference between the measured and simulated means is evident (0.184 mm), it is minor, confirming that the simulation model accurately predicts deformation behavior within a reasonable margin.

In summary, although the simulation underestimates the actual deformations by a small margin, it demonstrates high precision in predicting the behavior of the light-guide samples. Due to the process complexity and the set boundary conditions, the observed deformations are still relatively high, but the simulation model has proven to be a reliable tool for predicting and optimizing the final deformations of the test specimens.

## 4. Discussion

This study concentrated on the injection molding process for thick-walled polycarbonate light guides, specifically evaluating how different process parameters influence deformations and sink mark depth. While both defects were studied, the focus shifted primarily to deformations due to the challenges in accurately determining where the maximum sink mark would occur on the complex geometry of the light guide. Moreover, the effect of sink marks was determined to have less impact on the overall part functionality compared to deformations, which directly affect the optical performance and assembly of the light guide. Based on the DOE results, it can also be inferred that improvements in deformation would correlate with a reduction in sink marks.

Through simulations, it was identified that the most influential parameters affecting deformations were injection pressure, melt temperature, and their interaction. The higher melt temperature allowed for a smoother flow of the material, leading to reduced internal stresses, especially in thicker regions of the part. The interaction between injection pressure and melt temperature was also significant, as maintaining higher injection pressure ensured that the material reached all areas of the mold, while the high melt temperature prevented early solidification, reducing potential distortions. The importance of this combination lies in the way that higher melt temperatures improve material fluidity, promoting better packing during the molding phase.

Interestingly, mold temperature had a less significant role in influencing the final deformations, although its interaction with other factors was statistically relevant. Lower mold temperatures were effective in enhancing the cooling process, but the relatively minor influence suggests that mold temperature plays a secondary role in comparison to the melt and injection pressures. This aligns with the idea that maintaining an appropriate melt temperature is more critical to achieving dimensional stability, as it allows for controlled solidification without inducing excessive residual stresses.

In terms of sink-mark depth, similar trends were observed. The interaction between injection pressure and melt temperature was again a major influence. Sink marks tend to form when the outer material solidifies and the inner material continues to shrink, creating depressions on the surface. The combination of high injection pressure and high melt temperature helped to reduce these surface defects by ensuring a more even distribution of material across the mold cavity, thus compensating for shrinkage during the cooling phase.

Despite the simulation results showing a strong correlation between process parameters and defect reduction, the actual experimental results revealed some remaining deformation. Due to the boundary conditions, including the set process parameters, mold design, and material properties, this was the lowest achievable deformation. The fact that the simulations closely mirrored the experimental outcomes suggests that the simulation models were effective in predicting real-world behavior, although they may not capture all the complexities of the actual molding process. Factors such as mold imperfections, slight variations in material composition, or machine-specific characteristics could contribute to the discrepancies observed.

The decision to focus on deformations in the later stages of this research was based on the more critical nature of these defects for the light guide. Deformations can lead to significant optical distortions, affecting light transmission and the overall performance of the part, whereas sink marks, though visible, have less impact on functionality. The assumption that reducing deformations would also reduce sink marks seems reasonable since both defects are influenced by similar process conditions, such as injection pressure and melt temperature.

Future studies should aim to further refine the process settings by exploring intermediate values between the tested levels of injection pressure and melt temperature. This could provide more nuanced control over the deformation and sink mark formation, especially in regions of the part that are more sensitive to dimensional changes. Additionally, a more thorough evaluation of sink marks, possibly through advanced imaging techniques or non-destructive testing, would provide better insights into how these surface defects evolve during the molding process.

In conclusion, this study highlights the effectiveness of simulation-driven design in optimizing the injection molding process for thick-walled polycarbonate parts. While further refinements are needed, especially in managing sink marks, the findings offer valuable guidance for controlling deformations and ensuring high product quality.

## 5. Conclusions

This study has provided valuable insight into the injection-molding process for thick-walled polycarbonate light guides. Through the integration of Autodesk Moldflow 2024 simulations and experimental validation, critical process parameters, including injection pressure, melt temperature, and mold temperature, were identified as influencing factors on both deformations and sink-mark depths. These parameters directly impact the dimensional stability and final quality of the molded parts.

While the study initially aimed to examine both deformations and sink marks, it became clear that deformations had a more profound influence on the performance and quality of the light guide, particularly in the optical regions. The research demonstrated that increasing injection pressure and melt temperature, while maintaining a lower mold temperature, minimized deformations, and the simulations aligned closely with experimental outcomes. The discrepancy between predicted and measured deformations was minimal, which affirms the accuracy of the simulation tools under the defined boundary conditions.

Although deformations were minimized, the study highlighted that the inherent challenges of thick-walled designs and material behavior still resulted in noticeable deviations. However, these were effectively captured by the simulations, which suggests that while process adjustments are essential, certain limitations are intrinsic to the material properties.

The findings of this research provide a strong basis for further studies in the injection molding of complex optical components, with the potential to improve both theoretical understanding and practical applications in industrial settings. This work offers a framework for optimizing molding parameters and improving the overall quality of thick-walled polycarbonate components, particularly in optical applications.

## Figures and Tables

**Table 1:** Volumetric mesh parameters of molded part.

Parameter	Value	Default Edge Length [mm]	Simulated Deformation [mm]
Number of Elements	3,058,525	2.5	2.35
Connected Nodes	553,674	1.8	2.20
Maximum Element Aspect Ratio	17.48	1.2	2.15
Minimum Element Aspect Ratio	1.01	1.1	2.11
Average Element Aspect Ratio	3.19	1.0 (converged)	2.10
Volume of Tetra Elements	71.50 cm^3^	0.9	2.10
Maximum Dihedral Angle	174.9°	–	–

**Table 2:** Defined parameters of initial simulations.

Selected Simulation Parameters	
Injection Molded Material	PC Makrolon LED 2245 (Covestro AG, Leverkusen, Germany)
Mold Material	Tool Steel P20 (1.2311) (Meusburger Georg GmbH + Co KG, Wolfurt, Austria)
Coolant	Oil (10 L/min)
Coolant Channel Diameter	6 mm
Injection Molding Machine	Arburg Allrounder 470 E 1000-290 (Arburg GmbH + Co KG, Loßburg, Germany)
Melt Temperature Values	280 °C
Mold Temperature Values	100 °C
Ejection Temperature	130 °C
Filling Time	5 s
Injection Pressure	Automatically
Velocity/Pressure Switchover	at 99% of Cavity Volume Filled
Packing Pressure	80% of Defined Injection Pressure
Packing Time	10 s

**Table 3:** Summary of the simulation results.

Examined Parameter	Film Gate	Three-Point Gate	Unit
Fill Time	5.2	6.8	s
Occurrence of Weld Lines	Negligible	Significant	-
Effective Packing Time	3.5	2.7	s
Maximum Shear Rate	12,533	14,091	s^−1^
Maximum Shear Stress at Wall	0.71	1.10	MPa
Maximum Deflection	2.42	2.67	mm
Maximum Sink Mark Depth	0.44	0.52	mm

**Table 4:** Selected ranges of process parameters for initial injection molding.

Process Parameter	Value	Unit
Melt Temperature	250–300	°C
Mold Temperature	80–120	°C
Injection Pressure	45–65	MPa
Packing Pressure	80% of Injection Pressure	–
Packing Time	10	s
Melt-Flow Rate	14.7	cm^3^/s

**Table 5:** Input parameters and their specified levels.

Input Factor	Process Parameter	Test Level 1 (Min. Value)	Test Level 2 (Max. Value)	Unit
A	Injection Pressure	45	63	MPa
B	Mold Temperature	80	120	°C
C	Melt Temperature	250	300	°C

**Table 6:** DOE incorporating simulated responses for maximum deformations and sink-mark depth.

Run Order	Injection Pressure [MPa]	Mold Temperature [MPa]	Melt Temperature [MPa]	Maximal Deformation [mm]	Max. Sink-Mark Depth [mm]
1	45	80	250	0.636	0.366
2	65	80	250	0.512	0.362
3	45	120	250	0.626	0.361
4	65	120	250	0.481	0.367
5	45	80	300	0.774	0.398
6	65	80	300	0.649	0.342
7	45	120	300	0.771	0.402
8	65	120	300	0.622	0.345

**Table 7:** Comparison of the statistical significance of process parameters influencing deformation and sink mark depth.

Order of Statistical Significance	Deformation	Sink Mark Depth
1.	AC	AC
2.	A	A
3.	C	C
4.	B	Not significant

**Table 8:** Summary of optimized process parameters for minimizing deformation and sink-mark depth.

Evaluated Parameter	Value	Unit
Min. Deformation	2.108	[mm]
Min. Sink-Mark Depth	0.343	[mm]
Injection Pressure	65	[MPa]
Mold Temperature	80	[°C]
Melt Temperature	300	[°C]

## Data Availability

The original contributions presented in the study are included in the article. Further inquiries can be directed to the corresponding author.
